# Estimated nationwide impact of implementing a preemptive pharmacogenetic panel approach to guide drug prescribing in primary care in The Netherlands

**DOI:** 10.1186/s12916-019-1342-5

**Published:** 2019-06-14

**Authors:** P. C. D. Bank, J. J. Swen, H. J. Guchelaar

**Affiliations:** Department of Clinical Pharmacy and Toxicology, Leiden University Medical Center, P.O Box 9600, 2300 RC Leiden, The Netherlands

**Keywords:** Pharmacogenetics, Pharmacogenomics, Clinical practice, General practice, Community pharmacy

## Abstract

**Background:**

Pharmacogenetics (PGx) is currently implemented in hospitals to optimize therapy with high-risk drugs. However, many drugs with dosing recommendations from the Dutch Pharmacogenetics Working Group and the Clinical Pharmacogenetics Implementation Consortium are used in primary care. Actionable phenotypes for the genes covered in these guidelines are common with estimates ranging from 85 to 95% of the population carrying at least one actionable phenotype. The goal of this study was to estimate the clinical impact of implementation of an upfront panel-based pharmacogenetic screening for eight genes related to drugs used in primary care for 2016.

**Methods:**

For this study, dispensing data concerning first prescription for the period January 1–December 31, 2016, were combined with frequency data obtained in the “Implementation of Pharmacogenetics into Primary Care Project” (IP3) study to estimate the occurrence of actionable gene-drug pairs in daily practice in community pharmacies.

**Results:**

In 23.6% of all new prescriptions of 45 drugs (*n* = 856,002 new prescriptions/year), an actionable gene-drug interaction (GDI) was present according to the guidelines of the Dutch Pharmacogenetics Working Group. More importantly, these GDIs would result in a dose adjustment or switch to another drug in 5.4% of all new prescriptions.

**Conclusions:**

Consequently, with an anticipated near future where healthcare professionals will be regularly confronted with PGx test results, adjusting pharmacotherapy based on this information will become a routine task in healthcare.

**Electronic supplementary material:**

The online version of this article (10.1186/s12916-019-1342-5) contains supplementary material, which is available to authorized users.

## Introduction

The current use of prescription drugs is suboptimal. Many patients suffer from poor drug efficacy which in turn can lead to cessation of therapy or disease progression. Another significant portion of patients experience serious drug adverse events with possible hospitalization or even death as a result of the current one-size-fits-all approach [1, 2]. In recent decades, it has become clear that the interpatient variability in drug efficacy and toxicity can be (partially) explained by genetic variation between individuals [3, 4]. Therefore, in 2005, the Royal Dutch Pharmacists Association formed the Dutch Pharmacogenetics Working Group (DPWG) to aid in the personalization of pharmacotherapy based on an individual’s genetic makeup. The DPWG has created a set of evidence-based guidelines (*n* = 86) which have been fully integrated into the electronic drug prescribing and dispensing systems and are available through clinical decision support [5–7]. Currently, in The Netherlands, pharmacogenetics (PGx) testing is mostly performed for single gene-drug pairs. For example, testing for *DPYD* before starting therapy with capecitabine or 5-fluorouracil is routine care in The Netherlands and supported by a consistent body of evidence [8–10]. The impact of PGx in this drug-gene combination is considered high as DPD-deficient patients receiving a normal dose of capecitabine have a high risk for severe toxicities [8]. Nowadays, genotyping platforms allow for simultaneous characterization of multiple genes. This approach has been evaluated in multiple studies in secondary centers [11–16]. Results indicate that > 95% of all individuals carry at least one actionable phenotype when tested for a panel of up to 12 genes (including, e.g., *CYP2C9*, *CYP2C19*, *CYP2D6*, *SLCO1B1*, and *VKORC1*) [17–19]. This panel-based approach has also been favorably evaluated in a number of small pilot studies in a primary care setting [20–22].

Most drugs frequently prescribed in primary care are not considered high risk. However, the combination of a high prescription volume and a high frequency of actionable phenotypes of the associated genes may also have resulted in a high total impact on the population of patients [23, 24]. In this study, we set out to make a quantitative estimate of the potential impact of implementing PGx in primary care by calculating the number of new prescriptions in The Netherlands that comprise a gene-drug pair that is considered actionable by the DPWG. Additionally, we investigated the frequency of prescriptions where a change in choice of drug or dosage would have been required at the start of therapy as described in the guidelines of the DPWG based on a prediction of their genetic predicted phenotype.

## Methods

### Selection of drug-gene interactions and classification of therapeutic recommendations

The methodology for guideline development of the DPWG has been described in detail previously [5–7]. For this study, the DPWG guidelines were reviewed for drugs with an actionable therapeutic recommendation for a least one phenotype. Therapeutic recommendations were classified in no action, lower dose, higher dose, alternate drug, additional clinical monitoring of the patient, optional lower dose, optional higher dose, and a maximum dose threshold. Additionally, the guidelines were checked to see whether the therapeutic recommendations were dependent on patient characteristics such as age and concomitant use of other medication. For citalopram and escitalopram, the therapeutic recommendations are dependent on the age of the patient as a lower dose is recommended for patients ≥ 65 years of age [5, 6, 25]. Secondly, for *SLCO1B1* and atorvastatin, the therapeutic recommendation depends on concomitant use of a CYP3A4 inhibiting drug, i.e., amiodarone, verapamil, or diltiazem. Patients with a *SLCO1B1* 521TC or CC genotype and a CYP3A4 inhibitor are advised to switch to rosuvastatin or pravastatin, whereas in patients without a CYP3A4 inhibitor only increased monitoring for muscle pain is recommended.

### Source of nationwide prescription data

The Foundation of Pharmaceutical Statistics (Stichting Farmaceutische Kengetallen, SFK) collects data on dispensed drugs from ~ 95% of all the community pharmacies in The Netherlands [26]. To this end, patients are assigned an anonymous identification number that allows tracking within the participating community pharmacies [26]. To this end, patients are assigned an anonymous identification number that allows tracking within the participating community pharmacies [26]. For this study, dispensing data concerning first prescription for the period January 1–December 31, 2016, were obtained. First prescriptions in The Netherlands are defined by healthcare insurers as the dispensing of a drug that has not been used by the patient in the prior 365 days. For citalopram, escitalopram, and atorvastatin, additional information concerning age, and concomitant medication of the patients were also collected [5, 6, 25].

### Frequencies of genetic predicted phenotypes

To estimate the potential clinical impact of implementation of preemptive testing for a panel consisting of 8 genes (*CYP2C9*, *CYP2C19*, *CYP2D6*, *CYP3A5*, *DPYD*, *SLCO1B1*, *TPMT*, and *VKORC1*) related to drugs used in primary care, frequency data obtained in the “Implementation of Pharmacogenetics into Primary Care Project” (IP3) study were used as a representation of the Dutch population [24]. In short, for the IP3 study, 200 patients receiving a new prescription for a selection of 10 drugs with a known gene-drug interaction were genotyped for a panel of 8 genes and 40 genetic variants (see Additional file 1) using the Affymetrix Drug Metabolism Enzymes and Transporters (DMET) platform supplemented with a RT-PCR Taqman assay to determine the *CYP2D6* copy-number variation (CNV). The genetic test results were translated to actionable phenotypes (e.g., extensive/normal, intermediate, poor, or ultra-rapid metabolizer or EM, IM, PM, and UM respectively) according to the interpretation tables provided by the DPWG guidelines, and communicated to the general practitioner and pharmacist to perform genotype-guided dosing using clinical decision support [24]. A comparison to the Genome Of the NetherLands (GONL) dataset, containing 250 Dutch parent-offspring families, showed similar minor allele frequencies (MAF) for the selection of SNPs tested in the IP3 study. Similarly, the MAFs of the SNPs in the Caucasian subpopulation in the IP3 study was comparable to the European non-Finnish population in the gnomAD database. Furthermore, specifically for *CYP2D6*, similar frequencies of genetic predicted phenotypes were recently reported by compiling data from the Clinical Pharmacogenetics Implementation Consortium (CPIC) guidelines [27]. All these comparisons show that the population of patients in the IP3 study reflect the ethnic composition of the population in The Netherlands and the study population is a representative sample of the Dutch population [28, 29]. Additionally, the percentage of patients with at least one genetic variant in the tested multigene panel was comparable to previously reported PGx implementation projects [12, 14, 17–19].

### Estimating the clinical impact of PGx in primary care

Frequencies of actionable phenotypes for the eight genes from the IP3 were inferred for the Dutch population. Following this comparison, the genetic data were combined with the prescription data obtained from the SFK. After exclusion of drugs not approved in The Netherlands (warfarin) or primarily used in secondary care (5-fluorouracil, capecitabine, and tegafur), a selection of 45 drugs remained (see Table 1). This final selection was used to estimate the occurrence of actionable gene-drug pairs in daily practice in community pharmacies by multiplying the estimated frequency of actionable phenotypes for each gene with the observed first prescriptions of the related drugs (see Table 2).

**Table 1:** Number of incident prescriptions in 2016. An overview of the total number of new prescriptions for drugs with an actionable DPWG recommendation dispensed in Dutch pharmacies in 2016 that supply data to the SFK sorted to drug name

ATC	Drug name	No. of first-time prescriptions
B01AA07	ACENOCOUMAROL	49,934
N06AA09	AMITRIPTYLINE	98,750
N05AX12	ARIPIPRAZOLE	13,869
N06BA09	ATOMOXETINE	1987
C10AA05	ATORVASTATIN	111,840
C10BA05	ATORVASTATIN AND EZETIMIBE	1909
L04AX01	AZATHIOPRINE	6943
N06AB04	CITALOPRAM	56,580
N06AA04	CLOMIPRAMINE	7079
B01AC04	CLOPIDOGREL	98,709
R05DA04	CODEINE	519,728
N02AJ06	CODEINE AND PARACETAMOL	69,300
N06AA12	DOXEPIN	270
N06AB10	ESCITALOPRAM	24,454
A02BC05	ESOMEPRAZOLE	65,370
B01AA04	FENPROCOUMON	12,621
N03AB02	FENYTOINE	828
C01BC04	FLECAINIDE	13,605
N05AD01	HALOPERIDOL	51,217
N06AA02	IMIPRAMINE	988
A02BC03	LANSOPRAZOLE	1536
L01BB02	MERCAPTOPURINE	2598
C07AB02	METOPROLOL	194,724
C07BB02	METOPROLOL MET THIAZIDE	1908
M01AE52	NAPROXEN AND ESOMEPRAZOLE	673
N06AA10	NORTRIPTYLINE	20,717
A02BC01	OMEPRAZOLE	575,353
N02AA05	OXYCODONE	464,799
N02AA55	OXYCODONE AND NALOXONE	82
A02BC02	PANTOPRAZOLE	361,741
A02BD04	PANTOPRAZOLE, AMOXICILLINE, AND CLARITROMYCINE	21,768
N06AB05	PAROXETINE	27,018
N05AG02	PIMOZIDE	1060
C01BC03	PROPAFENON	409
N06AB06	SERTRALINE	28,861
C10AA01	SIMVASTATIN	187,362
C10BA02	SIMVASTATIN AND EZETIMIBE	4888
L04AD02	TACROLIMUS	2722
L02BA01	TAMOXIFEN	10,807
L01BB03	TIOGUANINE	1883
N02AX02	TRAMADOL	357,389
N02AJ13	TRAMADOL AND PARACETAMOL	124,951
N06AX16	VENLAFAXINE	26,603
J02AC03	VORICONAZOLE	891
N05AF05	ZUCLOPENTHIXOL	1873

**Table 2:** Overview of inferred gene-drug pairs sorted per gene. An overview of the estimates of the occurrences of gene-drug pairs among 45 drugs frequently prescribed in primary care

Drug	Count incident prescriptions (total)	Actionable phenotype	Count incident prescriptions	Type of therapeutic recommendation
*CYP2C9*				
PHENYTOIN*	828	EM	518	No action
		IM	294	Lower dose required at start therapy
		PM	17	Lower dose required at start therapy
*CYP2C19*				
CITALOPRAM < 65 years^*^	41,338	EM	29,557	No action
		IM	8,888	Guard maximum daily dose
		PM	1240	Guard maximum daily dose
		UM	1654	No action
CITALOPRAM ≥ 65 years^*^	15,242	EM	10,898	No action
		IM	3277	Guard maximum daily dose
		PM	457	Guard maximum daily dose
		UM	610	No action
CLOPIDOGREL^*^	98,709	EM	70,577	No action
		IM	21,222	Switch to alternate drug at start therapy
		PM	2961	Switch to alternate drug at start therapy
		UM	3948	Observe status of patient carefully
ESCITALOPRAM < 65 years^*^	21,427	EM	15,320	No action
		IM	4607	Guard maximum daily dose
		PM	643	Guard maximum daily dose
		UM	857	No action
ESCITALOPRAM ≥ 65 years^*^	3027	EM	2164	No action
		IM	651	Guard maximum daily dose
		PM	91	Guard maximum daily dose
		UM	121	No action
ESOMEPRAZOLE	65,370	EM	46,740	No action
		IM	14,055	No action
		PM	1961	No action
		UM	2615	Optional increase of dose
ESOMEPRAZOLE AND NAPROXEN	673	EM	481	No action
		IM	145	No action
		PM	20	No action
		UM	27	Optional increase of dose
LANSOPRAZOLE	1536	EM	1098	No action
		IM	330	No action
		PM	46	No action
		UM	61	Optional increase of dose
OMEPRAZOLE	575,353	EM	411,377	No action
		IM	123,701	No action
		PM	17,261	No action
		UM	23,014	Optional increase of dose
PANTOPRAZOLE	361,741	EM	258,645	No action
		IM	77,774	No action
		PM	10,852	No action
		UM	14,470	Optional increase of dose
PANTOPAC	21,768	EM	15,564	No action
		IM	4680	No action
		PM	653	No action
		UM	871	Higher dose required at start therapy
SERTRALINE^*^	28,861	EM	20,636	No action
		IM	6205	Guard maximum daily dose
		PM	866	Guard maximum daily dose
		UM	1154	No action
VORICONAZOLE^*^	891	EM	637	No action
		IM	192	Observe status of patient carefully
		PM	27	Observe status of patient carefully
		UM	36	Higher dose required at start therapy
*CYP2D6*				
AMITRIPTYLINE^*^	98,750	EM	52,338	No action
		IM	39,994	Switch to alternate drug at start therapy
		PM	4938	Switch to alternate drug at start therapy
		UM	1481	Switch to alternate drug at start therapy
ARIPIPRAZOLE	13,869	EM	7351	No action
		IM	5617	No action
		PM	693	Guard maximum daily dose
		UM	208	No action
ATOMOXETINE^*^	1987	EM	1053	No action
		IM	805	Optional decrease of dose
		PM	99	Optional decrease of dose
		UM	30	Observe status of patient carefully
CLOMIPRAMINE^*^	7079	EM	3752	No action
		IM	2867	Lower dose required at start therapy
		PM	354	Lower dose required at start therapy
		UM	106	Switch to alternate drug at start therapy
CODEINE^*^	519,728	EM	275,456	No action
		IM	210,490	No action
		PM	25,986	No action
		UM	7796	Switch to alternate drug at start therapy
CODEINE AND PARACETAMOL^*^	69,300	EM	36,729	No action
		IM	28,067	Optional increase of dose
		PM	3465	Switch to alternate drug at start therapy
		UM	1040	Switch to alternate drug at start therapy
DOXEPIN^*^	270	EM	143	No action
		IM	109	Lower dose required at start therapy
		PM	14	Lower dose required at start therapy
		UM	4	Switch to alternate drug at start therapy
FLECAINIDE	13,605	EM	7211	No action
		IM	5510	Lower dose required at start therapy
		PM	680	Lower dose required at start therapy
		UM	204	Observe status of patient carefully
HALOPERIDOL	51,217	EM	27,145	No action
		IM	20,743	No action
		PM	2561	Lower dose required at start therapy
		UM	768	Optional increase of dose
IMIPRAMINE (TOTAL)	988			
IMIPRAMINE (CYP2C19 EM, IM, UM)^*^	958	EM	508	No action
		IM	388	Lower dose required at start therapy
		PM	48	Lower dose required at start therapy
		UM	14	Switch to alternate drug at start therapy
IMIPRAMINE (CYP2C19 PM) ^*^	30	EM	16	Lower dose required at start therapy
		IM	12	Lower dose required at start therapy
		PM	2	Lower dose required at start therapy
		UM	0	Switch to alternate drug at start therapy
METOPROLOL	194,724	EM	103,204	No action
		IM	78,863	Optional decrease of dose
		PM	9736	Optional decrease of dose
		UM	2921	Optional increase of dose
METOPROLOL AND THIAZIDE	1908	EM	1011	No action
		IM	773	Optional decrease of dose
		PM	95	Optional decrease of dose
		UM	29	Optional increase of dose
NORTRIPTYLINE^*^	20,717	EM	10,980	No action
		IM	8390	Lower dose required at start therapy
		PM	1036	Lower dose required at start therapy
		UM	311	Switch to alternate drug at start therapy
OXYCODONE	464,799	EM	246,343	No action
		IM	188,244	Observe status of patient carefully
		PM	23,240	Observe status of patient carefully
		UM	6972	Observe status of patient carefully
OXYCODONE AND NALOXONE	82	EM	43	No action
		IM	33	Observe status of patient carefully
		PM	4	Observe status of patient carefully
		UM	1	Observe status of patient carefully
PAROXETINE^*^	27,018	EM	14,320	No action
		IM	10,942	No action
		PM	1351	No action
		UM	405	Switch to alternate drug at start therapy
PIMOZIDE	1060	EM	562	No action
		IM	429	Lower dose required at start therapy
		PM	53	Lower dose required at start therapy
		UM	16	No action
PROPAFENON	409	EM	217	No action
		IM	166	Optional decrease of dose
		PM	20	Lower dose required at start therapy
		UM	6	Observe status of patient carefully
TAMOXIFEN^*^	10,807	EM	5728	No action
		IM	4377	Switch to alternate drug at start therapy
		PM	540	Switch to alternate drug at start therapy
		UM	162	No action
TRAMADOL^*^	357,389	EM	189,416	No action
		IM	144,743	Optional increase of dose
		PM	17,869	Optional increase of dose
		UM	5361	Lower dose required at start therapy
TRAMADOL AND PARACETAMOL^*^	124,951	EM	66,224	No action
		IM	50,605	Optional increase of dose
		PM	6248	Optional increase of dose
		UM	1874	Lower dose required at start therapy
VENLAFAXINE	26,603	EM	14,100	No action
		IM	10,774	Switch to alternate drug at start therapy
		PM	1330	Switch to alternate drug at start therapy
		UM	399	Optional increase of dose
ZUCLOPENTHIXOL	1873	EM	993	No action
		IM	759	Lower dose required at start therapy
		PM	94	Lower dose required at start therapy
		UM	28	Optional increase of dose
*CYP3A5*				
TACROLIMUS^*^	2722	Non-Ex	2314	No action
		Het-Ex	395	Higher dose required at start therapy
		Homo-Ex	14	Higher dose required at start therapy
*SLCO1B1*				
ATORVASTATIN (TOTAL)	111,840			
ATORVASTATIN (WITHOUT CYP3A4 INHIBITOR)	108,400	NT (521TT)	80,758	No action
		PT (521TC)	25,474	Observe status of patient carefully
		PT (521CC)	2168	Observe status of patient carefully
ATORVASTATIN (WITH CYP3A4 INHIBITOR)	3440	NT (521TT)	2563	No action
		PT (521TC)	808	Switch to alternate drug at start therapy
		PT (521CC)	69	Switch to alternate drug at start therapy
ATORVASTATIN AND EZETIMIBE	1909			
ATORVASTATIN AND EZETIMIBE (WITHOUT CYP3A4 INHIBITOR)	1739	NT (521TT)	1296	No action
		PT (521TC)	409	Observe status of patient carefully
		PT (521CC)	35	Observe status of patient carefully
ATORVASTATIN AND EZETIMIBE (WITH CYP3A4 INHIBITOR)	170	NT (521TT)	127	No action
		PT (521TC)	40	Switch to alternate drug at start therapy
		PT (521CC)	3	Switch to alternate drug at start therapy
SIMVASTATIN^*^	187,362	NT (521TT)	139,585	No action
		PT (521TC)	44,030	Switch to alternate drug at start therapy
		PT (521CC)	3747	Switch to alternate drug at start therapy
SIMVASTATIN AND EZETIMIBE^*^	4888	NT (521TT)	3642	No action
		PT (521TC)	1149	Switch to alternate drug at start therapy
		PT (521CC)	98	Switch to alternate drug at start therapy
*TPMT*				
AZATHIOPRINE^*^	6943	EM	5867	No action
		IM	1041	Lower dose required at start therapy
		PM	35	Switch to alternate drug at start therapy
MERCAPTOPURINE^*^	2598	EM	2195	No action
		IM	390	Lower dose required at start therapy
		PM	13	Switch to alternate drug at start therapy
TIOGUANINE^*^	1888	EM	1591	No action
		IM	282	Lower dose required at start therapy
		PM	9	Switch to alternate drug at start therapy
*VKORC1*				
ACENOCOUMAROL	49,934	NS (1173CC)	16,478	No action
		NS (1173CT)	25,217	No action
		HS (1173TT)	8239	Lower dose required at start therapy
FENPROCOUMON	12,621	NS (1173CC)	4165	No action
		NS (1173CT)	6374	No action
		HS (1173TT)	2082	Lower dose required at start therapy

^*^Gene-drug interactions with a recommendation both by the DPWG and CPIC

*EM* extensive/normal metabolizer, *IM* intermediate metabolizer, *PM* poor metabolizer, *UM* ultra-metabolizer, *Non-Ex* non-expressor, *Het* heterozygous expressor, *HOM* homozygous expressor, *NT* normal transport activity, *PT* poor transport activity, *NS* normal sensitivity, *HS* high sensitivity

## Results

**Fig. 1:**
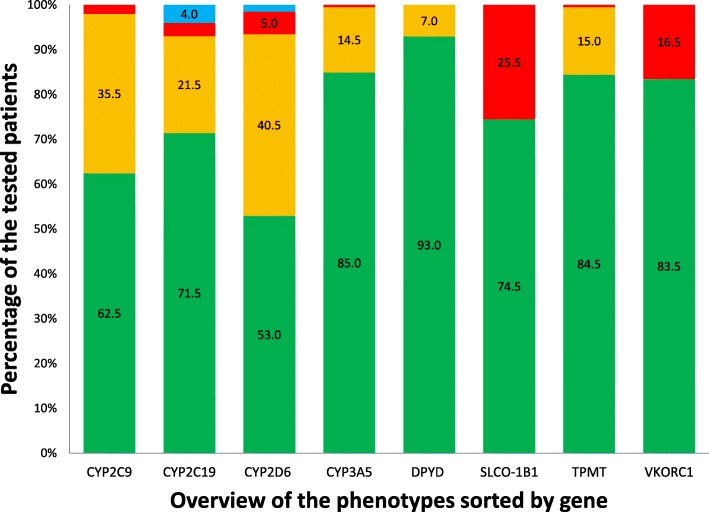
An overview of the genotype predicted phenotypes observed in the IP3 pilot study. Green: extensive/normal metabolizer (*CYP2C9, CYP2C19, CYP2D6, DPYD & TPMT*) / non-expressor (*CYP3A5*) / normal transporter activity (*SLCO1B1*) / normal sensitivity (*VKORC1*). Orange: intermediate metabolizer (*CYP2C9, CYP2C19, CYP2D6, DPYD & TPMT*) / heterozygous expressor (*CYP3A5*). Red: poor metabolizer (*CYP2C9, CYP2C19, CYP2D6, DPYD & TPMT*) / homozygous expressor (*CYP3A5*) / poor transporter activity (*SLCO1B1*) / high sensitivity (*VKORC1*). Blue: ultra-rapid metabolizer (*CYP2C19 & CYP2D6*) (see Additional file 1 for an overview of the tested variants)

For this analysis, prescription data for the selection of 45 drugs were available from 1882 pharmacies (94.4% of total). In 2016, a total of 3,338,464 unique patients received a total of 3,628,597 new prescriptions for the selected 45 drugs (see Table 1). The distribution of the phenotypes for the eight genes is presented in Fig. 1. Based on the frequencies of the actionable phenotypes of the eight genes and the amount of 3,628,597 first prescriptions, it can be estimated that in 856,002 new prescriptions (23.6%) a gene-drug pair is present (see Table 2). In 195,691 new prescriptions (= 5.4%), the gene-drug pair requires an action by the prescribing physician or dispensing pharmacists. These actions include lowering the dose (*n* = 43,616), increasing the dose (*n* = 1315), or switching to an alternate drug (*n* = 150,761) as per the recommendations of the DPWG. For the remainder of the prescriptions where an actionable gene-drug pair is present (*n* = 660,311), no direct action is required; however, more intensified monitoring for side effects is recommended (*n* = 250,980), guarding a lower maximum threshold of the prescribed dose designated by the DPWG for specific phenotypes (*n* = 26,924) or a potential decrease (*n* = 90,543) or increase (*n* = 291,863) of the dose in case of observed over- or undertreatment respectively (see Table 2).

In case all patients treated in primary care received pre-emptive screening for the panel of eight genes, ~ 54 per 1000 first prescriptions would have required an intervention for the selected 45 drugs.

Additionally, healthcare professionals would be required to monitor patients more intensively for side effects or failure of treatment in ~ 236 per 1000 first prescriptions of the drugs included in the selection.

This results in a calculated number needed to genotype of 19 for a required intervention on a first time prescription by a healthcare professional at the start of therapy for this selection of 45 drugs. Of note, this calculated number needed to genotype is likely a conservative estimate as interventions for gene-drug interactions (23.6%) of the “monitor closely” type were not used to calculate the number needed to genotype as it is currently unknown how many interventions are performed in this type of gene-drug interaction.

### *CYP2C19* and (es)citalopram

For the selective serotonin reuptake inhibitors (SSRI) citalopram, escitalopram, and sertraline, the DPWG has provided a lower maximum dose for intermediate and poor metabolizers compared to extensive/normal metabolizers [5, 6, 25]. However, in contrast to sertraline, the maximum dosages for citalopram and escitalopram have alternate thresholds for patients < 65 years of age vs. ≥ 65 years. For example, for a patient with a first prescription for citalopram with a poor metabolizer phenotype < 65 years, the advised maximum daily dose is 20 mg/day, whereas for a patient ≥ 65 years with the same phenotype the advised maximum daily dose is 10 mg/day.

In 41,338 of the 56,580 new prescriptions for citalopram (= 73.1%), the patient was below the age of 65 whereas in 15,424 cases the patient was 65 years or older (= 26.9%). From these data, it can be inferred that in 8888 prescriptions, a patient with an IM phenotype of age under 65 should have received a dose restriction of 30 mg and in 3277 prescriptions a patient with an IM phenotype of age 65+ should have been restricted to a maximum dose of 15 mg. For PMs, the dose should have been maximized to 20 mg in individuals of < 65 years old (*n* = 1240 prescriptions) and 10 mg for age 65+ (*n* = 457 prescriptions) respectively.

In case of escitalopram, the dose restriction should have been 15 mg (*n* = 4607 prescriptions) and 7.5 mg (*n* = 651 prescriptions) for patients with the IM phenotype in the groups < 65 and ≥ 65 respectively. In case of 643 patients younger than 65 years of age with the PM phenotype, the dose should have been restricted to 10 mg, whereas in 91 patients of ≥ 65 years with the same inferred phenotype the dose should have been restricted to 5 mg (see Table 2).

### *SLCO1B1* and atorvastatin

The frequency of patients with a reduced transport capacity mediated as a result of genetic variation in the *SLCO1B1* gene was found to be 25.5% of which 23.5% was the result of the 521T/C genotype and 2.0% the result of a 521C/C genotype. In 2016, a total of 113,749 first prescriptions for atorvastatin or atorvastatin combined with ezetimibe were dispensed. Based on the sample of genotype data from the IP3 study, we extrapolated that in 29,006 prescriptions the patient would likely carry the 521T/C or the 521C/C genotype. For these individuals, an additional check for concomitant use of inhibitors of CYP3A4 is required as per the DPWG guidelines and in case of the presence of a CYP3A4 inhibitor healthcare professionals are recommended to switch to an alternate drug.

## Discussion

This study provides an estimate of the potential nationwide clinical impact of a pre-emptive pharmacogenetic panel approach in primary care in The Netherlands. Based on frequencies of actionable phenotypes gathered in a pilot study of 200 patients and nationwide prescription data (*n* = 3,628,597), we inferred that in 23.6% of the first prescriptions of the selected 45 drugs a gene-drug pair is present [24]. If pre-emptive genetic testing of the panel consisting of eight genes had been performed in this population, we estimate that 5.4% of the new prescriptions an intervention at the start of therapy would have been required and in 18.2% of the new prescriptions the therapeutic recommendations of the DPWG advise more intense clinical monitoring of the patient with an optional dose adjustment in case of suboptimal therapy. An example of such a recommendation is the optional increase in dose of a proton pump inhibitor in a *CYP2C19* ultra-rapid metabolizer.

A strength of this study is that the dataset used to calculate the frequency of gene-drug pairs compasses 1882 pharmacies (94.4% of total) and reflects all community pharmacies in The Netherlands [26]. This allows for a detailed view of the medication use of the total population treated in primary care and provides accurate data of the number of first prescriptions of one of the PGx drugs where patients have not yet reached their maintenance therapy as a result of empirical dosing by general practitioners. In a similar study by Samwald et al., prescription data from a combination of sources (private insurance, Medicaid or Medicare) were used [30]. Whereas the dataset used in that study encompasses a considerable larger number of patients, it is at risk for selection bias as it is restricted to selected groups within the population of the USA [30].

Although the data in this analysis encompasses 94.4% of the pharmacies in The Netherlands and provides a detailed view of drug use in primary care, other sections of pharmaceutical care are not represented in this dataset. A majority of the 5.6% of pharmacies that do not supply data to the database of the SFK are mainly outpatient pharmacies (community pharmacies as part of a hospital). In comparison with community pharmacies, the outpatient pharmacies often dispense more specialized pharmacotherapy, for example HIV therapy. For example, this may result in a higher frequency of drug-drug interactions through CYP3A4 and higher frequency of relevant drug-drug-gene interactions for statins. The number of first prescriptions where a patient uses a CYP3A4 inhibitor may likely be an underestimation of the reality.

Another limitation to this study is the lack of complete clinical data (such as comorbidities, reduced clearance of drugs, and information on indications) in the available dataset. For a limited number of gene-drug interactions, the therapeutic recommendation of the DPWG is co-dependent on these clinical data and co-medication. Unfortunately, our dataset only contained information about age, gender, and co-medication with CYP3A4 inhibitors. As a result, estimates for gene-drug interaction requiring interpretation of additional clinical factors or information about indication to support decision making might differ from reality. For example, in case of the interaction of simvastatin and the *SLCO1B1* 521TC genotype, the primary recommendation is to switch to an alternate therapy (used in this analysis to infer therapeutic interventions). However, if the prescribed dose is ≤ 40 mg simvastatin, the recommendation is to continue simvastatin provided the patient carries no additional risk for statin-induced myopathy. Additional risk factors include hepatic or renal impairment, co-medication with CYP3A4 inhibitors, co-medication with SLCO1B1 inhibitors, female gender, old age (≥ 65) or hypothyroidism [7, 25]. To estimate the potential impact of clinical factors on the number of patients requiring an intervention, we conducted a sensitivity analyses for these risk factors (excluding co-medication with SLCO1B1 inhibitors) for both simvastatin and atorvastatin.

Based on the genotype frequencies inferred from the IP3 study and the SFK prescription data, out of all the first-time prescriptions for simvastatin, 44,030 individuals carried the *SLCO1B1* 521TC genotype (see Table 2). Using publicly available data on prevalence of relevant co-factors, we estimate that ~ 10,000 males with the 521TC genotype without any risk factors are present where the recommendation would be to continue therapy with 40 mg simvastatin [31, 32]. A similar sensitivity analysis was carried out for the patients receiving simvastatin with ezetimibe. The initial analysis identified 1149 patients who would require a switch based on their inferred *SLCO-1B1* 521TC genotype (see Table 2). Using the same sensitivity analysis used for simvastatin, we estimate approximately 266 individuals with the 521TC genotype do not carry additional risk factors and do not require a change in therapy.

For atorvastatin, the data presented in Table 2 may be an underestimation of the trough number of switches required in clinical practice. In contrast to simvastatin, the consideration of clinical co-factors would result in additional switches. The sensitivity analysis for atorvastatin identified 6160 male patients without additional risk factors who are recommended to start atorvastatin with the normal dose from the total 27,642 patients with 521TC and CC genotypes. In contrast, in a group of ~ 21,000 patients, atorvastatin should be switched to an alternate therapy due to the existence additional risk factors identified by the DPWG. A similar analysis for the combination of atorvastatin with ezetimibe shows 83 patients with the 521TC or CC genotype without risk factors who can start therapy with the ezetimibe/atorvastatin combination, while in 289 patients with these genotypes additional risk factors are present likely resulting in a switch to an alternate cholesterol-lowering therapy. Overall, the results of both our sensitivity analysis indicate that without accounting for additional clinical factors for the gene-drug interactions concerning *SCLO1B1*, our estimates of 195,691 patients requiring an intervention is conservative and could be as high as ~ 206,981.

Additionally, due to the structure of the obtained prescription data, the analysis in this study was performed from the perspective of prescriptions within the time frame of a year. The IP3 study (used for this analysis) and multiple other studies using panel-based pharmacogenetic testing show that patients often carry multiple actionable pharmacogenes [17–19, 24]. In reality, physicians and pharmacists will thus likely encounter multiple actionable phenotypes in the same patient and due to polypharmacy are likely to encounter multiple gene-drug interactions in each patient [33]. Additional follow-up of the IP3 study indeed showed that within 2.5-year follow-up, 97% of the included patients received a prescription for a newly initiated drug with an actionable DGI, while 33% of the patients received up to four new prescriptions with known gene-drug interactions. In the newly started prescriptions in our cohort, 24.2% of the patients indeed carried the actionable phenotype, requiring an intervention by the pharmacist [34]. These data show that an increase in the studied timeframe will show a larger impact on healthcare. However, our results indicate that pre-emptive PGx testing for a panel of eight genes can already have significant impact on first prescriptions within the timeframe of a single year. Results of such a PGx test can be re-used over the entire lifespan of an individual. It is highly likely that impact will be even larger in reality as 13.5% of the patients that visit (Dutch) community pharmacies use ≥ 5 drugs from different ATC3 classes [26]. Moreover, due to the increase in the proportion of elderly (≥ 65 years), the number of polypharmacy patients will likely increase in the future, increasing the possible impact of a pharmacogenomics panel [26].

As a result of the use of a PGx panel with a limited number of pharmacogenes, this may lead to an underestimate of the potential impact of PGx in primary care in The Netherlands. After the initiation of the IP3 pilot study, the DPWG has published additional guidelines on gene-drug pairs other than *CYP2C9, CYP2C19, CYP2D6, CYP3A5, DPYD, SLCO1B1, TPMT*, and *VKORC1* existing on the DMET micro-array platform but not included in this panel (e.g., *CYP1A2*, *CYP2B6*, and *UGT1A1*) [24]. In addition, the chosen platform in the pilot study does not encompass all genes deemed relevant by the DPWG such as *FVL* and *HLA* genes. Of note, this study only encompasses interventions based on therapeutic recommendations for gene-drug interactions described in the guidelines of the DPWG as they are incorporated in the clinical decision support systems of healthcare professionals in The Netherlands. In clinical practice, the Dutch clinicians will thus most likely only use the therapeutic recommendations by the DPWG. In countries where the guidelines of the CPIC are used to guide pharmacotherapy, the number of prescriptions with a combination of actionable phenotypes and associated drugs will likely differ somewhat as the DPWG has provided therapeutic recommendations for certain gene-drug interactions that are not covered by CPIC and vice versa. However, a recent systematic comparison of the guidelines of the CPIC and the DPWG found that a selection of 27 well-known gene-drug interactions (all included in the selection of this study) was covered by both consortia and the two consortia provided similar therapeutic recommendations for these gene-drug interactions [7].

Similar to the Ubiquitous Pharmacogenomics (U-PGx) project, current genotyping initiatives using a pre-emptive panel approach should use a platform which provides an extensive panel and is flexible (such as the QuantStudio 12K Flex Real-Time PCR System by Thermo Fisher Scientific or the SNPline by LGC Group) to allow for adjusting if new guidelines are published by CPIC or the DPWG [35].

Currently, the implementation of PGx in primary care remains low in The Netherlands as shown by a recent survey among Dutch pharmacists that showed 14.7% adoption of PGx testing, despite the existence of guidelines containing therapeutic recommendations and their integration into the workflow of healthcare professionals [5, 6, 36]. In part, this may be explained by the fact that not all healthcare professionals are aware of the existence of clinical decision support in electronic medication surveillance systems. A recent survey among pharmacists showed that 65.4% were aware that clinical decision support contained therapeutic recommendations for genetic predicted phenotypes [36]. The potential of PGx has grown, but probably other healthcare professionals (such as physicians) are less aware of the state in which PGx is already available in their workflow. Finally, the clinical utility and cost-effectiveness of pre-emptive panel-based screening for PGx variants has only been shown in small pilot studies, but large clinical trials providing the required evidence are still lacking [20–22]. Initiatives such as the U-PGx, a multinational consortium implementing a model of pre-emptive PGx-testing in healthcare environments in seven European nations, will investigate whether upfront implementation of a panel-based pharmacogenetic screening can lead to better patient outcomes in a cost-effective manner [35].

## Conclusions

Based on the data presented in this study, it can be concluded that an actionable gene-drug interaction is present in approximately one out of four new prescriptions for a drug classified by the DPWG guidelines as requiring therapeutic intervention. Should all patients with a new prescription for this selection of drugs have been pre-emptively genotyped, 1 out of every 19 new prescriptions could have been adjusted based on the genetic test result. Consequently, with an anticipated near future where healthcare professionals will be confronted with results of PGx panels, adjusting pharmacotherapy as a result of relevant gene-drug interaction will likely become a routine task in drug prescribing.

## Additional file

Additional file 1: Overview of tested PGx variants. Description of data: An overview of the PGx variants included in the panel used in the Implementation of Pharmacogenetics into Primary Care Project (IP3) study. (DOCX 16 kb)
