# Supplementary material for: Estimated nationwide impact of implementing a preemptive pharmacogenetic panel approach to guide drug prescribing in primary care in The Netherlands
Source: BMC Med. 2019 Jun 14;17:110. doi: 10.1186/s12916-019-1342-5 (PMC6567386; doi:10.1186/s12916-019-1342-5)
Supplement: Supplementary file 1 — Overview of tested PGx variants. Description of data: An overview of the PGx variants included in the panel used in the Implementation of Pharmacogenetics into Primary Care Project (IP3) study. (DOCX 16 kb) [file 12916_2019_1342_MOESM1_ESM.docx]

# Additional file 1 – Overview of tested PGx variants

| Gene | Allele | Reference Sequence + Variant | RS-number |
| --- | --- | --- | --- |
| CYP2C9 | *2 | NG_008385.1:g.3608C>T | rs1799853 |
| CYP2C9 | *3 | NG_008385.1:g.42614A>C | rs1057910 |
| CYP2C19 | *2 | NG_008384.3:g.19154G>A | rs4244285 |
| CYP2C19 | *3 | NG_008384.3:g.17948G>A | rs4986893 |
| CYP2C19 | *17 | NG_008384.3:g.-806C>T | rs12248560 |
| CYP2D6 | *2A | M33388:g.-1584C>G | rs1080985 |
| CYP2D6 | *10 | M33388:g.100C>T | rs1065852 |
| CYP2D6 | *12 | M33388:g.124G>A | rs5030862 |
| CYP2D6 | *11 | M33388:g.883G>C | rs201377835 |
| CYP2D6 | *17 | M33388:g.1023C>T | rs28371706 |
| CYP2D6 |  | M33388:g.1661G>C | rs1058164 |
| CYP2D6 | *6 | M33388:g.1707delT | rs5030655 |
| CYP2D6 | *4 | M33388:g.1846G>A | rs3892097 |
| CYP2D6 | *40 | M33388:g.1863_1864insTTTCGCCCCTTTCGCCCC | rs72549356 |
| CYP2D6 | *20 | M33388:g.1973_1974insG | rs72549354 |
| CYP2D6 | *19 | M33388:g.2539delAACT | rs72549353 |
| CYP2D6 | *3 | M33388:g.2549delA | rs35742686 |
| CYP2D6 | *38 | M33388:g.2587delGACT | rs72549351 |
| CYP2D6 | *9 | M33388:g.2615delAAG | rs5030656 |
| CYP2D6 |  | M33388:g.2850C>T | rs16947 |
| CYP2D6 | *7 | M33388:g.2935A>C | rs5030867 |
| CYP2D6 | *44 | M33388:g.2950G>C | rs72549349 |
| CYP2D6 | *41 | M33388:g.2988G>A | rs28371725 |
| CYP2D6 | *29 | M33388:g.3183G>A | rs59421388 |
| CYP2D6 | *42 | M33388:g.3259_3260insGT | rs72549346 |
| CYP2D6 | *18 | M33388:g.4132_4133insGTGCCCACT | rs1135836 |
| CYP2D6 |  | M33388:g.4180G>C | rs1135840 |
| CYP2D6 | *5 | NC_000022.10:g.[0] |  |
| CYP2D6 | xN | duplication |  |
| CYP3A5 | *3 | NG_007938.1:g.12083G>A | rs776746 |
| CYP3A5 | *6 | NG_007938.1:g.19787G>A | rs10264272 |
| DPYD | *2A | NM_000110.3:c.1905+1G>A | rs3918290 |
| DPYD | *13 | NM_000110.3:c.1679T>G | rs55886062 |
| DPYD |  | NM_000110.3:c.1236G>A | rs56038477 |
| DPYD |  | NM_000110.3:c.2846A>T | rs67376798 |
| SLCO1B1 |  | NM_006446.4:c.521T>C | rs4149056 |
| TPMT | *2 | NM_000367.4:c.238G>C | rs1800462 |
| TPMT | *3B | NM_000367.4:c.460G>A | rs1800460 |
| TPMT | *3C | NM_000367.4:c.719A>G | rs1142345 |
| VKORC1 |  | NM_206824.2:c.173+1000C>T | rs9934438 |
